# Harnessing NKG2D CAR-T cells with radiotherapy: a novel approach for esophageal squamous cell carcinoma treatment

**DOI:** 10.3389/fimmu.2025.1589379

**Published:** 2025-05-29

**Authors:** Tianyu Liu, Liyuan Fan, Weicheng Huang, Pengxiang Chen, Yuchen Liu, Shuyun Wang, Kaiyue Guo, Yufeng Cheng, Yali Han

**Affiliations:** ^1^ Department of Radiation Oncology, Qilu Hospital of Shandong University, Jinan, Shandong, China; ^2^ Laboratory of Basic Medical Sciences, Qilu Hospital of Shandong University, Jinan, Shandong, China; ^3^ Shandong Provincial Key Laboratory of Malignant Tumor Precision Treatment, Cancer Institute of Shandong University, Jinan, Shandong, China; ^4^ Phase I Clinical Trail Center, Shandong Cancer Hospital and Institute, Shandong First Medical University and Shandong Academy of Medical Sciences, Jinan, Shandong, China

**Keywords:** NKG2D, CAR-T cells, radiotherapy, combination treatment, ESCC

## Abstract

**Background:**

Esophageal squamous cell carcinoma (ESCC) represents a highly aggressive malignancy with poor prognosis and limited therapeutic advancements. While chimeric antigen receptor (CAR)-T-cell therapy has revolutionized cancer treatment, its application in ESCC remains poorly explored. This study pioneers the exploration of natural killer group 2 member D (NKG2D) CAR-T cells combined with radiotherapy for treating ESCC, with the goals of establishing a novel treatment strategy and achieving superior tumor control through combined immunoradiotherapy

**Methods:**

Flow cytometry and quantitative real-time PCR (qRT-PCR) were carried out to evaluate the expression of NKG2D ligands at the cell surface protein and mRNA levels. Cell-based bioluminescence assays and enzyme-linked immunosorbent assays (ELISAs) were performed to measure the cytotoxicity and cytokine secretion of NKG2D CAR-T cells. A human ESCC subcutaneous xenograft model and a bilateral xenograft model were established. Luminex liquid suspension chip detection was utilized to verify the changes in cytokines and chemokines in the circulation and at tumor sites. Immunohistochemical analysis was conducted to assess the accumulation of T cells *in vivo*.

**Results:**

NKG2D ligands are widely expressed in ESCC cell lines and can be further increased by irradiation at both the mRNA and cell surface protein levels. NKG2D CAR-T cells efficiently recognized and lysed ESCC cell lines, and irradiation enhanced the activity of NKG2D CAR-T cells targeting ESCC cells. Additionally, NKG2D CAR-T cells specifically homed to and accumulated in ESCC tumors, exerting efficient immunological activity correlated with noticeable tumor regression in a human ESCC xenograft model, with no obvious toxicity. Moreover, preconditioning with local radiotherapy accelerated the tumor shrinkage induced by NKG2D CAR-T cells, explaining by altering the tumor microenvironment (TME) and promoting the migration and infiltration of CAR-T cells into tumor sites.

**Conclusions:**

We first clarified the therapeutic efficacy of NKG2D CAR-T cells in ESCC, as well as their enhanced effect when combined with radiotherapy, which provides a novel treatment strategy for ESCC patients.

## 1 Introduction

Esophageal cancer is a highly aggressive malignancy and ranks as the sixth leading cause of cancer-related death and the seventh leading cause of cancer incidence worldwide (1). Esophageal cancer presents with two major histopathological forms, esophageal squamous cell carcinoma (ESCC) and esophageal adenocarcinoma (EAC), with ESCC accounting for 90% of all diagnosed patients (1). Despite significant advances in multimodal treatment regimens, including surgery, radiotherapy, chemotherapy, and immunotherapy, long-term survival rates for esophageal cancer patients remain unsatisfactory, with a relative overall five-year survival rate of only 20–30% (2). Radiotherapy is a standard treatment option for locally advanced esophageal cancer, preoperative therapy for resectable disease, and definitive or palliative therapy for unresectable cases. However, some patients cannot undergo full-dose radiotherapy because of the high risk of radiation toxicity (2, 3). Thus, novel treatment options or optimal combination treatment with radiotherapy are urgently needed for patients with ESCC.

NKG2D (natural killer group 2, member D) is an activating immune receptor associated with tumor immunosurveillance, normally expressed on all NK cells, CD8+ T cells, subsets of γδT cells, and some autoreactive CD4+ T cells (4). The molecular structure of NKG2D allows its binding to several ligands in humans, including the MHC I chain-related molecules A and B (MICA and MICB) and a family of six UL16-binding proteins (ULBP1-6). NKG2D ligands (NKG2DLs) are typically not found or are minimally expressed in healthy tissues but are widely expressed in malignant cells, serve as immune surveillance markers that allow the immune system to recognize (4–6). The engagement of NKG2D by NKG2DLs activates cytotoxic effect in immune cells, leading to production of cytokines and target cell lysis (4). However, tumors have evolved multiple mechanisms to evade NKG2D-mediated immune surveillance, such as epigenetic silencing of NKG2DL expression or the shedding of soluble NKG2DLs, which can downregulate NKG2D receptors on immune cells and impair their function (4, 5). Therefore, NKG2D introduced into chimeric antigen receptor (CAR) engineered T cells have been explored as a promising immunotherapy approach for the NKG2DLs-expressing tumors. In recent years, we and others have demonstrated the antitumor efficacy of NKG2D CAR-T cells in xenograft models of leukemia (7), liver cancer (8), gastric cancer (9, 10), colorectal cancer (11), ovarian cancer (12), breast cancer (13), glioma (14), osteosarcoma (15), prostate cancer (16) and pancreatic cancer (17). In addition, a few clinical trials testing NKG2D CAR-T cells are currently ongoing, and preliminary data are promising (18). However, no NKG2D CAR has been reported for ESCC treatment.

The application of NKG2D CAR-T cell therapy for ESCC is supported by the finding that MICA and MICB, family members of NKG2DLs, are significantly overexpressed in ESCC cells compared with adjacent normal tissues and that NKG2DLs are widely expressed on the surface of ESCC cell lines (19). In addition, NKG2DLs can be induced by various cellular stress stimuli, such as malignant cell transformation or DNA damage, and their expression in tumor cells can be further increased when they are exposed to different doses of radiation (20–22). Moreover, radiation has the potential to modify the immunosuppressive tumor microenvironment (TME) in solid tumors, thereby enhancing the antitumor immune response to immunotherapy (23). These findings provide a strong rationale for combining NKG2D CAR-T cells therapy with radiotherapy for ESCC treatment.

Therefore, we constructed an NKG2D-BBz CAR for ESCC and evaluated its antitumor activities as well as its enhanced effect when combined with radiation *in vitro* and in xenograft mouse models. Here, we demonstrate for the first time that NKG2DL-expressing ESCC is sensitive to NKG2D CAR-T cells attack. Furthermore, we found that radiation upregulated the expression of various NKG2DLs in ESCC cell lines and improved the potency of NKG2D CAR-T cells-mediated attack *in vitro* and in a human ESCC xenograft model, suggesting that the combination of NKG2D CAR-T cells therapy and radiotherapy could be a promising treatment modality for ESCC.

## 2 Materials and methods

### 2.1 Cell lines and culture

Eca109, Kyse150, and TE‐1 were sourced from the China Center for Type Culture Collection (Wuhan University, Wuhan, China). Kyse140 and Kyse510 were obtained from Professor Yan Li of the Sun Yat-sen University Cancer Center (Guangzhou, China). HEK293T cells were obtained from the Chinese Academy of Sciences (Shanghai, China). Tumor cells were modified to express firefly luciferase (fLuc) for bioluminescence assays. The cells were cultured in RPMI-1640 medium (Gibco BRL, USA) enriched with 10% FBS (Gibco), 100 U/ml penicillin, and 100 mg/ml streptomycin in a 37 °C incubator with a humidified atmosphere and 5% CO_2_.

### 2.2 CAR construction and lentivirus production

The targeting domain, which consists of the extracellular domain (ECD) of human NKG2D or anti-CD19 scFv, was synthesized by Genechem (Shanghai, China) and cloned into a CAR-encoding lentiviral backbone containing a human CD8 hinge spacer and transmembrane domain, 4-1BB, and CD3ζ endodomains. CARs were inserted into the GV401 plasmid (Genechem, China) and expressed under the control of the EF1a promoter. For lentiviral packaging, HEK293T cells were cotransfected with 20 µg of lentiviral plasmid, 15 µg of pHelper 1.0 and 10 µg of pHelper 2.0 packaging plasmids (Genechem, China). After 48 h of transfection, the supernatants were harvested and then concentrated for 2 h at 25000 rpm using a Beckman XE-90 rotor (Beckman Coulter) through ultracentrifugation. The copy number of the lentiviral particles was confirmed using qRT-PCR.

### 2.3 Human T cells and transfection

T cell samples were collected from the peripheral blood of four healthy donors using an approved protocol by the Medical Ethical Committee of Qilu Hospital, Shandong University (approved ID: KYLL-81902919). We obtained written informed consent from each donor in accordance with the Declaration of Helsinki. Lymphocyte separation medium (TBD, LTS1077) was used to isolate primary T cells following the manufacturer’s protocol. Afterwards, the T cells were stimulated with CD3/CD28 beads (Gibco, 11131D) under a bead-to-cell ratio of 3:1 for 24 h and then infected with lentiviral particles at a multiplicity of infection (MOI) of 5. We maintained the density of T cells between 0.5 × 10^6^ and 1 × 10^6^ cells/ml during a two-week expansion period. Human recombinant interleukin-2 (IL-2; from PeproTech, USA) was added to reach a final concentration of 100 IU/mL. T cells were allowed to rest in medium without IL-2 for at least 24 h before being utilized in subsequent assays (24).

### 2.4 Cytotoxicity assays

For the cell-based bioluminescence assays, 1 × 10^4^ fLuc-expressing tumor cells were cocultured with T cells at effector-to-target (E/T) ratios of 1:1, 2:1, 4:1, or 8:1 in a 96-well microplate (Beyotime, RG052M). For blocking experiments, transduced T cells were preincubated with anti-NKG2D or isotype antibody from Invitrogen for 2 h at 37°C. For irradiation pretreatment *in vitro*, ESCC cells were exposed to 6 MV X-rays (Clinac-23-EX, Varian, USA) at the Radiation Centre of Qilu Hospital 48 h before coincubation with T cells. Single fractions of 2, 4, and 8 Gy were administered. After 20 h of coculture, each well was filled with 100 µl of Bright-Lumi™ firefly luciferase reporter gene detection reagent and then tested for chemiluminescence. Enzyme-linked immunosorbent assays (ELISAs) were conducted to assess the concentrations of perforin and granzyme B in the supernatant of the coculture system using kits from Zci Bio (ZC-32647; ZC-33834). The values presented are the means of triplicate wells.

### 2.5 Cytokine secretion assays

Assays for cytokine secretion were conducted in a coculture system with T cells and tumor cells at an E/T ratio of 4:1 in 96-well flat-bottom microplates containing 200 µl of medium. The supernatants were collected after a 20-hour coculture, and the levels of tumor necrosis factor (TNF)-α, interferon (IFN)-γ, interleukin (IL)-10, and interleukin (IL)-2 were measured via ELISA kits (BD Biosciences, 550612, 550613, 550610, 550611) according to the manufacturer’s protocols. The presented data represent the means obtained from triplicate wells.

### 2.6 Flow cytometric analysis

The cells were harvested, washed, and resuspended at a concentration of 1 × 10^6^ cells/ml. The labeled primary antibodies were then introduced into the cell suspension and incubated in darkness at 4°C for 30 min. The purity of the isolated T cells was assessed utilizing a PE-conjugated anti-human CD3 antibody (BioLegend, 300307), and NKG2D expression in the transfected T cells was analyzed using a PE/Cyanine5-conjugated anti-human CD314 (NKG2D) antibody (BioLegend, 320844). APC-anti-MICA (R&D System, FAB1300A), APC-anti-MICB (R&D System, FAB1599A), APC-anti-ULBP1 (R&D System, FAB1380A), APC-anti-ULBP2/5/6 (R&D System, FAB1298A), APC-anti-ULBP3 (R&D System, FAB1517A), and APC-anti-ULBP4 (R&D System, FAB6285A) were utilized to analyze NKG2DL expression on the surface of ESCC cells. As controls, matched isotype antibodies were employed in each analysis. For irradiated tumor cells, dead cells were excluded from the analysis of NKG2DL expression. Flow cytometry analysis was executed on the BD FACS Calibur+Sort platform. The data analysis was carried out with FlowJo software.

### 2.7 Quantitative real-time PCR

Total RNA was extracted from ESCC cell lines using RNA Fast 2000 (Fastagen), and its concentration was quantified using a NanoDrop spectrophotometer. Samples with A260/A280 ratios between 1.8 and 2.2 and A260/A230 ratios between 2.0 and 2.5 were deemed pure. The minimum RNA concentration for downstream analysis was 50 ng/µl. RNA was stored at -80°C until use. An Evo M-MLV RT Premix for qPCR Kit (Accurate Biotechnology, AG11706) was used to perform reverse transcription. The synthesized cDNA was stored at -20°C for short-term use or -80°C for long-term preservation. Quantitative real-time PCR was conducted in triplicate to evaluate gene expression using SYBR Green from Accurate Biotechnology. The following primers were used.

MICA: forward 5’-AGGGTTTCTTGCTGAGGTACA-3’,
reverse 5’-GGTCTCTCTGTCCCATGTCTTA-3’,
MICB: forward 5’-TCTTCGTTACAACCTCATGGTG-3’,
reverse 5’-TCCCAGGTCTTAGCTCCCAG-3’,
ULBP1: forward 5’-TAAGTCCAGACCTGAACCACA-3’,
reverse 5’-TCCACCACGTCTCTTAGTGTT-3’,
ULBP2: forward 5’-GTGGTGGACATACTTACAGAGC-3’,
reverse 5’-CTGCCCATCGAAACTGAACTG-3’,
ULBP3: forward 5’-TCTATGGGTCACCTAGAAGAGC-3’,
reverse 5’-TCCACTGGGTGTGAAATCCTC-3’,
ULBP4: forward 5’-GCACTTGGGGAGAATTGACCC-3’,
reverse 5’-ACATCTCGACTTGCAGAGTGG-3’.
Human GAPDH was used as a reference gene:
forward 5’- GGACCTGACCTGCCGTCTAG-3’,
reverse 5’- GTAGCCCAGGATGCCCTTGA-3’.

The conditions were as follows: 1 cycle at 95°C for 30 s and 40 cycles at 95°C for 5 s and 60°C for 30 s. All the results were normalized to those of GAPDH and calculated via the ▵▵Ct method for relative quantification.

### 2.8 Xenograft model of ESCC

Four- to five-week-old NOD/ShiLtJGpt-Prkdcem26Cd52Il2rgem26Cd22/Gpt (NCG) female mice were purchased from Gempharmatech Co. Ltd. The mice were kept in a controlled specific pathogen-free (SPF) facility with regulated temperature, humidity, and lighting. A total of thirty-eight mice were used in this study. To establish a human ESCC xenograft model, fLuc-expressing Eca109 tumor cells were subcutaneously inoculated into the flanks of NCG mice on day 0 (5 × 10^6^ cells per injection per mouse). The mice were randomly assigned to different groups via a randomization table to minimize potential bias. The growth of the tumors and the health of the mice were monitored continuously. Primary human T cells were amplified *in vitro* for two weeks, and then administered to mice via the tail vein on days 7 and 14 after tumor implantation (1x10^7^ cells/100 µl per injection per mouse). Bioluminescence signals were measured approximately every ten days. In the combined treatment groups, the mice were exposed to 8 Gy of additional radiation (X-Rad225 OptiMAX, PXi), and the rest of the body was shielded with a 3 mm thick lead plate. In the short-term observation cohort, euthanasia was performed and tumors, peripheral blood, and other organs were collected five days after T cell infusion. The long-term monitoring cohort were euthanized when tumors reached a predefined endpoint or on day 62 post-tumor implantation, whichever occurred first. Part of the tumor tissue was used for immunohistochemical staining, and the other part was used to extract proteins for multiple cytokine evaluation using Luminex liquid suspension chip detection (Wayen Biotechnologies, China). All mice were euthanized by intraperitoneal injection of pentobarbital sodium (150–200 mg/kg) following the designated time points for each group. All animal procedures received approval from the Animal Care and Use Committee of Shandong University and were carried out according to the Guidelines for Animal Health and Use (Ministry of Science and Technology, China, 2006).

### 2.9 Bioluminescence imaging

Tumor growth was monitored through bioluminescence imaging with a Xenogen IVIS imaging system (IVIS Spectrum, PerkinElmer). After subcutaneous tumor formation, the mice bearing fLuc-expressing Eca109 tumors were anesthetized with 2.5% avertin by intraperitoneal injection (15 ml/kg) followed by and intraperitoneally injected with IVISbrite D-Luciferin potassium salt bioluminescent substrate (PerkinElmer, 122799) suspended in PBS (15 mg/ml stock solution, 10 µl/g mouse body weight), and fluorescence imaging was performed 10 min later. Pseudocolor images representing light intensity were generated from living images.

### 2.10 Immunohistochemistry analysis

The tissue samples were embedded in paraffin, sectioned at 4 μm, deparaffinized, and rehydrated, followed by antigen retrieval. This was followed by peroxidase blocking with H2O2 (3%) for 20 min and a 1-hour blocking step using 10% serum from the same species as the source of the secondary antibody. CD3 recombinant rabbit monoclonal antibody (HUABIO, HA720082) was subsequently applied to the sections at 4°C overnight at a dilution ratio of 1:100. The sections were then incubated with a horseradish peroxidase (HRP)-conjugated secondary antibody. DAB (ZSGB-Bio) was used for immunohistochemical staining, and hematoxylin was used for counterstaining. The distributions of human T cells in mouse tumors and in the heart, lung, stomach, liver, spleen, pancreas, and kidney were observed.

### 2.11 Statistical analysis

The *in vitro* experiments were repeated at least three times. Statistical analysis was performed via GraphPad Prism 8.0, and the data are presented as the means and standard deviations (SDs). Two-tailed Student’s t tests were used to evaluate the significant differences between two groups. When there were three or more groups, significant differences were determined via one-way ANOVA and Sidak correction. The Kaplan–Meier method was utilized for survival analysis, while differences in survival time were determined by the log-rank test. Statistical significance was defined as * P < 0.05, ** P < 0.01, and *** P < 0.001.

## 3 Results

### 3.1 NKG2DLs are widely expressed in ESCC cells and increased by irradiation

**Figure 1:**
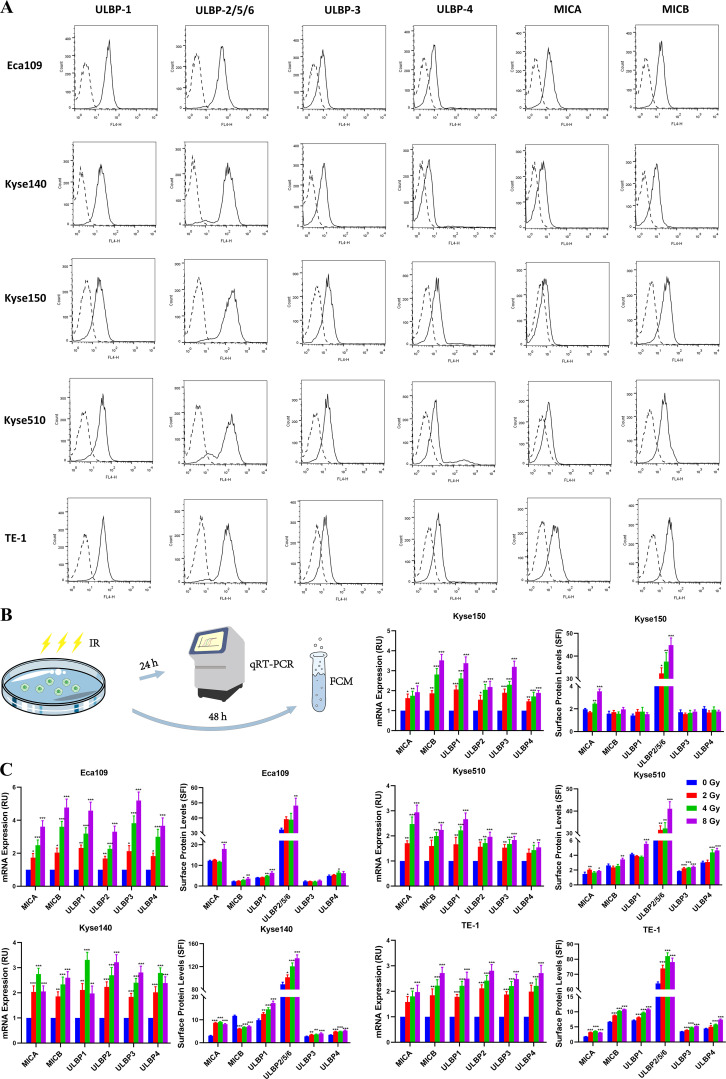
Expression of NKG2D ligands in ESCC cell lines. **(A)** A panel of human ESCC cell lines (Eca109, Kyse140, Kyse150, Kyse510, and TE-1) were stained with specific antibodies recognizing MICA, MICB, ULBP-1, ULBP-2/5/6, ULBP-3, or ULBP-4 (solid line histogram) or matched isotype antibodies (dashed line histogram) for flow cytometry. Specific fluorescence indices (SFIs) were calculated by dividing the median fluorescence obtained with the specific antibody by the median fluorescence obtained with the isotype control antibody. **(B, C)** Eca109, Kyse140, Kyse150, Kyse510, and TE-1 cell lines were exposed to 0, 2, 4 and 8 Gy irradiation, respectively. Transcripts (MICA, MICB, ULBP1, ULBP2, ULBP3 and ULBP4) were determined by qRT-PCR 24 h after irradiation. The NKG2DL protein levels on the cell surface were measured via flow cytometry 48 h after irradiation. *P < 0.05, **P < 0.01, ***P < 0.001.

To investigate the expression levels of NKG2DLs in human ESCC, we conducted flow cytometry analyses on five ESCC cell lines utilizing antibodies recognizing MICA, MICB, ULBP-1, ULBP-2/5/6, ULBP-3, or ULBP-4. As expected, we observed varying levels of NKG2DL expression on the surface of all the ESCC cell lines, ranging from high to moderate, across the six NKG2DL family members (**Figure 1A**). All the ESCC cell lines expressed moderate to low levels of MICA/B, and only Kyse150 cells presented very low levels of MICA. The levels of ULBPs expression in ESCC cell lines were heterogeneous. ULBP1 and ULBP 2/5/6 were highly expressed in most ESCC cell lines, whereas ULBP-3 and ULBP-4 were more often expressed at moderate-to-low levels. NKG2DLs appear to be widely expressed in ESCC cells, and only a few ligands in individual cell lines are expressed at relatively low levels.

Next, we evaluated the potential effects of irradiation on NKG2DL expression in ESCC cell lines. Eca109, Kyse140, Kyse150, Kyse510, and TE-1 cell lines were exposed to different doses of irradiation (0, 2, 4, or 8 Gy). Transcripts of MICA, MICB, ULBP1, ULBP2, ULBP3, and ULBP4 were assessed via qRT-PCR, and surface protein levels were determined via flow cytometry (**Figure 1B**). All five ESCC cell lines showed high sensitivity to radiation, with the mRNA level of NKG2DLs significantly increased in irradiated tumor cells compared with unirradiated cells 24 h after irradiation, and surface protein levels were upregulated approximately 48 h after irradiation (**Figure 1C**). A clinically relevant single dose within the 2–4 Gy range was adequate for the induction of NKG2DL expression.

### 3.2 NKG2D CAR-T cells efficiently recognize and lyse ESCC cell lines *in vitro*

**Figure 2:**
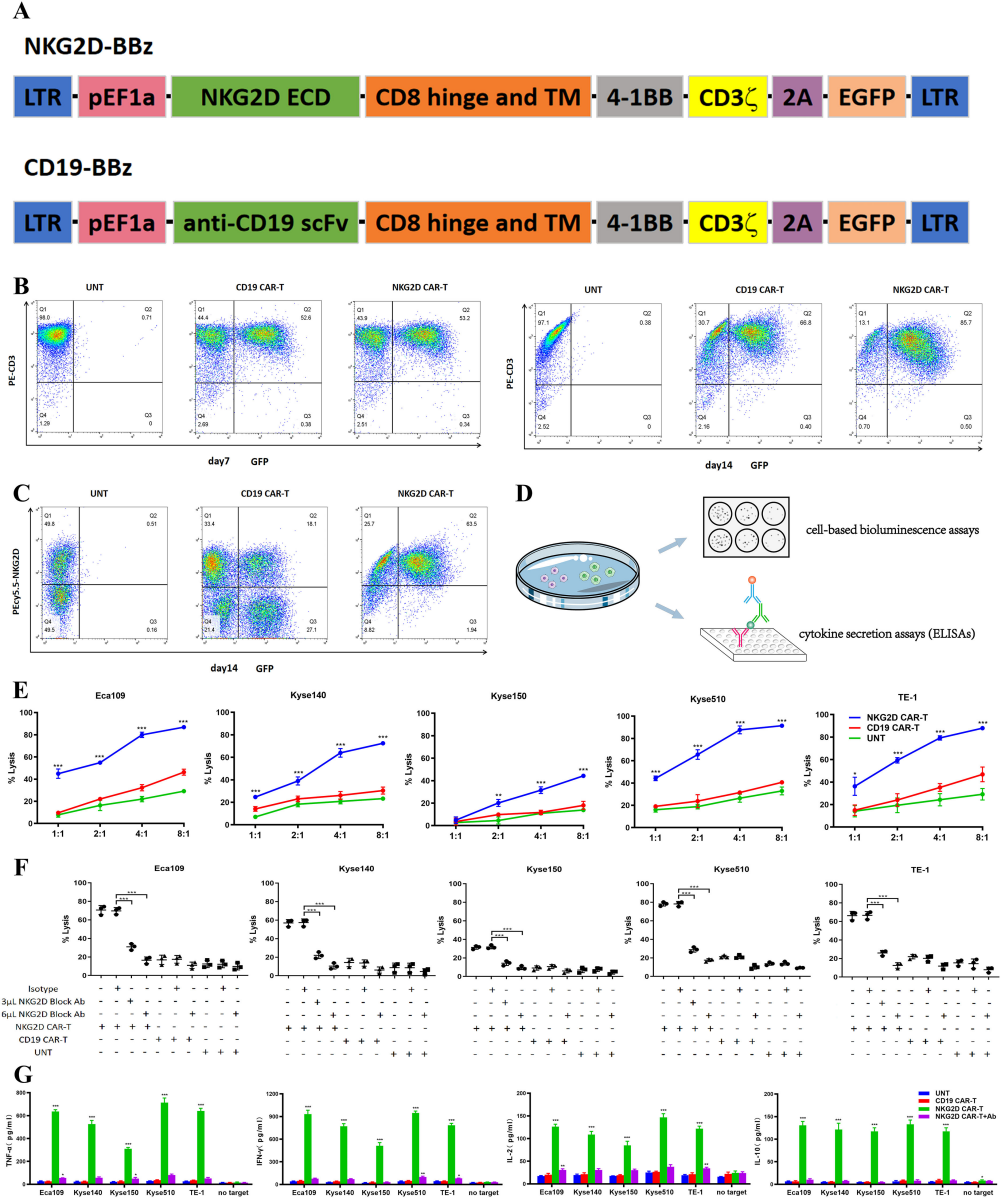
Recognition and lysis of ESCC cells by NKG2D CAR-T cells *in vitro*. **(A)** Schematic illustration of the construct for NKG2D-BBz CAR and CD19-BBz CAR. **(B)** The transduction efficiency of NKG2D CAR and CD19 CAR were analyzed by flow cytometry on day7 and day14 after transduction. The NKG2D CAR-T cells are enriched over time. **(C)** The endogenous NKG2D expression on NKG2D CAR-T, CD19 CAR-T and UNT cells were stained with anti-NKG2D antibodies and analyzed by flow cytometry. **(D)** Experimental design diagram of evaluating the cytotoxicity and recognition ability of T cells to ESCC cells *in vitro*. **(E)** NKG2D CAR-T, CD19 CAR-T and UNT cells were cocultured with the Eca109, Kyse140, Kyse150, Kyse510, and TE-1 cell lines respectively, at varying E:T ratios for 20 hours. Cell killing was determined by a standard bioluminescence cytotoxic assay. *P < 0.05, **P < 0.01, ***P < 0.001. **(F)** NKG2D CAR-T, CD19 CAR-T and UNT cells were preincubated with NKG2D-blocking antibodies (3 µl or 6 µl) or isotype control antibodies 2 hours before cocultured with ESCC cells at an E:T ratio of 4:1. Cell killing was determined as described above. ***, P < 0.001. **(G)** Cytokines (TNF-α, IL-10, IL-2 and IFN-γ) in the supernatant of the coculture system were detected by ELISA. NKG2D CAR-T, CD19 CAR-T and UNT cells were cocultured with Eca109, Kyse140, Kyse150, Kyse510, and TE-1 cell lines at an E:T ratio of 4:1. NKG2D-blocking antibodies were introduced into the NKG2D CAR-T cells coculture system as a control. T cells alone (no target) were used as a blank control. ***, P < 0.001.

The widely expression of NKG2DLs in ESCC cells supports the use of NKG2D CAR-T cells as a potential therapy for ESCC. NKG2D CARs were developed, consisting of an extracellular domain (ECD) of the human NKG2D receptor linked to a CD8α hinge and transmembrane region, followed by an intracellular 4-1BB costimulatory domain and a CD3ζ signaling motif (NKG2D-BBz). The GFP sequence was incorporated into bicistronic expression vectors to confirm and detect CAR transduction in T cells. A similar CD19-specific CAR using an anti-CD19 scFv was designed as an antigen-specific control (CD19-BBz) for the assays (**Figure 2A**).

T cells were activated utilizing beads coated with anti-CD3/CD28 antibodies and subsequently transduced with lentiviruses encoding NKG2D-BBz and CD19-BBz. Flow cytometry was conducted to evaluate CAR expression on days 7 and 14 post transduction. The percentage of T cells positive for the NKG2D CAR and CD19 CARs on day 7 was ~50%, whereas the percentage of NKG2D CAR-positive T cells was highly enriched to ~85% at the end of 14 days expansion (**Figure 2B**), which is consistent with a previous report that NKG2DLs expressed on activated T cells mediate fratricide but enrich for NKG2D CAR-T cells (12). In addition, we detected endogenous NKG2D expression on NKG2D CAR-T, CD19 CAR-T, and untransduced T (UNT) cells (**Figure 2C**), in accordance with the activation-induced upregulation of endogenous NKG2D known to occur in human T lymphocytes (25).

*In vitro*, we evaluated the cytotoxic capacity of T cells against ESCC cells via a cell-based bioluminescence assay (**Figure 2D**). Compared with CD19 CAR-T and UNT cells, NKG2D CAR-T cells efficiently lysed all ESCC cells in a dose-dependent manner. The cytotoxicity of NKG2D CAR-T cells increased with increasing E:T ratios. At an E:T ratio of 1:1, NKG2D CAR-T cells displayed a cytotoxicity of approximately 40% toward the Eca109, Kyse510, and TE-1 cell lines. The cytotoxicity increased to over 80% as the E:T ratio reached 4:1. Kyse140 and Kyse150 cells were less sensitive to NKG2D CAR-T cells owing to lower expression of NKG2DLs (**Figure 2E**). When we introduced NKG2D-blocking antibodies into the coculture system, NKG2D CAR-T cells failed to induce specific lysis of ESCC cells, with cytotoxicity reduced to baseline levels, suggesting that the cytotoxic effect of engineered T cells was NKG2D construct dependent (**Figure 2F**).

To further determine the recognition of NKG2DLs on ESCC cells by NKG2D CAR-T cells, the release of cytokines in the coculture medium was assessed via ELISAs (**Figure 2D**). NKG2D CAR-T cells specifically recognized NKG2DL-positive ESCC cells and secreted high levels of TNF-α, IFN-γ, IL-10, and IL-2 in overnight cocultures, while no response was observed in the absence of target tumor cells (**Figure 2G**). The level of cytokine response generally tends to be associated with the level of NKG2DL expression on the target cell surface. CD19 CAR-T and UNT cells did not secrete cytokines in response to any ESCC cells (**Figure 2G**). When introducing NKG2D blocking antibody to the co-culture system, the cytokines release by NKG2D CAR-T cells reduced to background levels, further verifying that the immunological effects exerted by NKG2D CAR-T cells can therefore be attributed specifically to NKG2D signaling and NKG2DLs interaction.

### 3.3 Radiation enhances the activity of NKG2D CAR-T cells against ESCC cells *in vitro*

**Figure 3:**
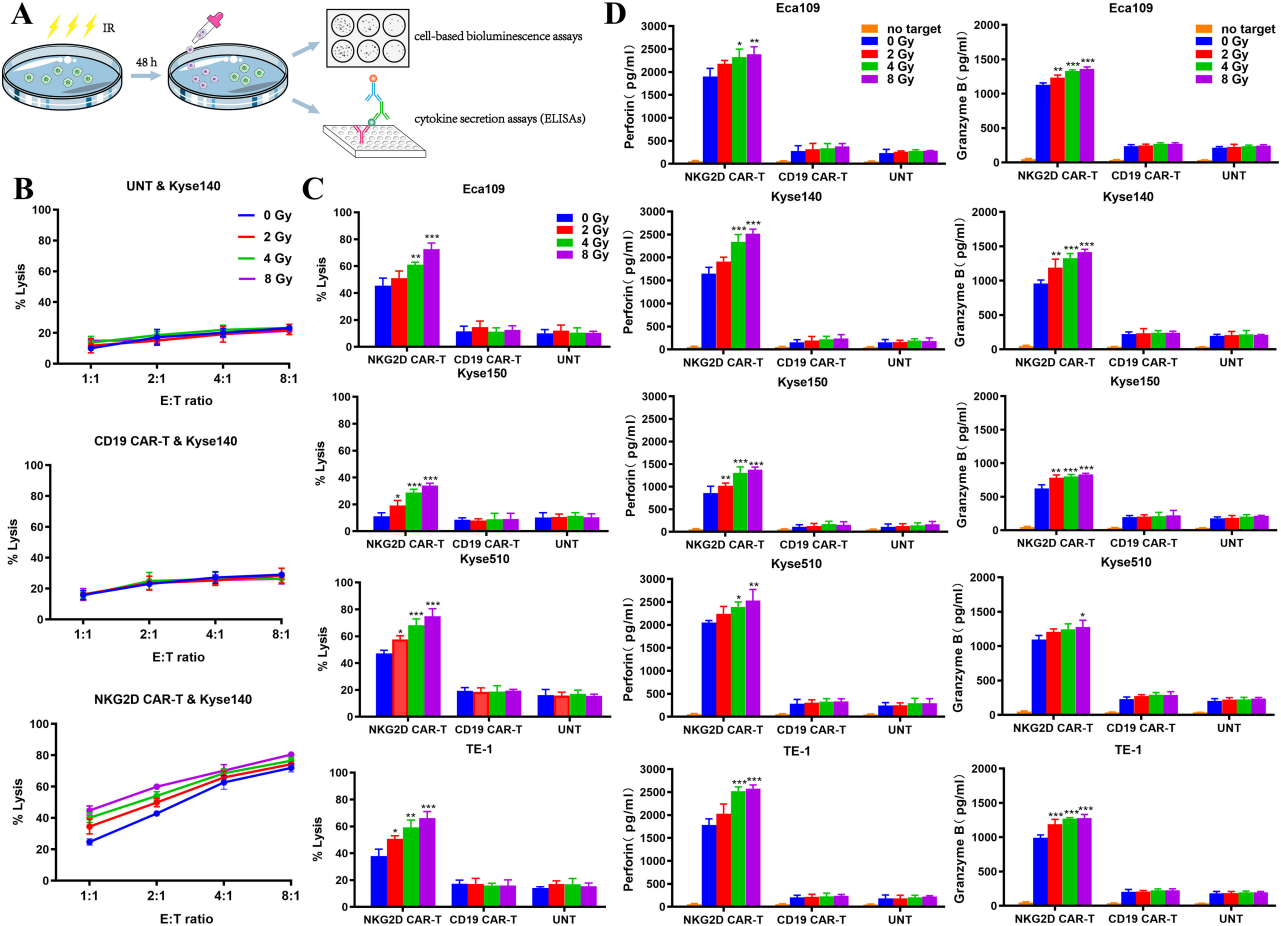
Characterization of NKG2D CAR-T cells toward preirradiated ESCC cells *in vitro*. **(A)** Experimental design diagram of evaluating the activity of NKG2D CAR-T cells against preirradiated ESCC cells *in vitro*. **(B)** The cytotoxicity of NKG2D CAR-T, CD19 CAR-T and UNT cells against preirradiated Kyse140 cells at different E:T ratios were measured by bioluminescence assays. Kyse140 cells were preirradiated with 2, 4, 8 Gy or not 48 hours before cocultured with T cells. **(C)** The cytotoxicity of NKG2D CAR-T, CD19 CAR-T and UNT cells against preirradiated Eca109, Kyse150, Kyse510 and TE-1 cells at a given E:T ratio of 1:1 was measured by bioluminescence assay. *P < 0.05, **P < 0.01, ***P < 0.001. **(D)** The levels of perforin and granzyme B in the supernatant of the coculture system described above were detected by ELISA. *P < 0.05, **P < 0.01, ***P < 0.001.

On the basis of the experimental results described above, we explored the effects of irradiation on the activity of NKG2D CAR-T cells in an *in vitro* coculture system. Kyse140 cells, as target cells, were pretreated with 0, 2, 4, or 8 Gy irradiation 48 h before cocultured with different T cells at E:T ratios of 8:1, 4:1, 2:1, or 1:1. Cell viability was measured via a bioluminescence assay, and preirradiated tumor cells were used as controls to eliminate background cell death induced by radiation (**Figure 3A**). Compared with nonirradiated cells, NKG2D CAR-T cells exerted greater cytotoxic effects toward preirradiated Kyse140 cells, and the cytotoxicity also increased with increasing E:T ratios (**Figure 3B**). As well, enhanced cytolytic activity of NKG2D CAR-T cells was observed toward preirradiated Eca109, Kyse150, Kyse510 and TE-1 cells under a given E:T ratio of 1:1 (**Figure 3C**). A single fraction of 2 Gy irradiation was sufficient to enhance the *in vitro* cytotoxicity of NKG2D CAR-T cells toward ESCC cells, while 8 Gy irradiation induced higher susceptibility of ESCC cells to NKG2D CAR-T cells attack compared to 2–4 Gy irradiation. The increase of cytolytic activity with increasing radiation dose was observed only in NKG2D CAR-T cells but not in CD19 CAR-T and UNT cells (**Figures 3B, C**).

The specific cytotoxicity of T cells is often indicated by their release of perforin and granzyme B when targeting tumor cells. Accordingly, we measured their concentrations in the supernatant of our coculture system by ELISAs (**Figure 3A**). The perforin and granzyme B concentrations in the NKG2D CAR-T coculture system were elevated following pretreatment of ESCC cells with increasing doses of irradiation, which was in line with increased tumor cell lysis. However, the concentrations of perforin and granzyme B in the supernatants of the CD19 CAR-T and UNT coculture systems did not vary with irradiation pretreatment (**Figure 3D**). This finding indicated that the increased cell mortality rate was due to the improved cytolytic activity of NKG2D CAR-T cells rather than radiation damage.

### 3.4 NKG2D CAR-T cells show effective and persistent antitumor activity against a human ESCC xenograft model

**Figure 4:**
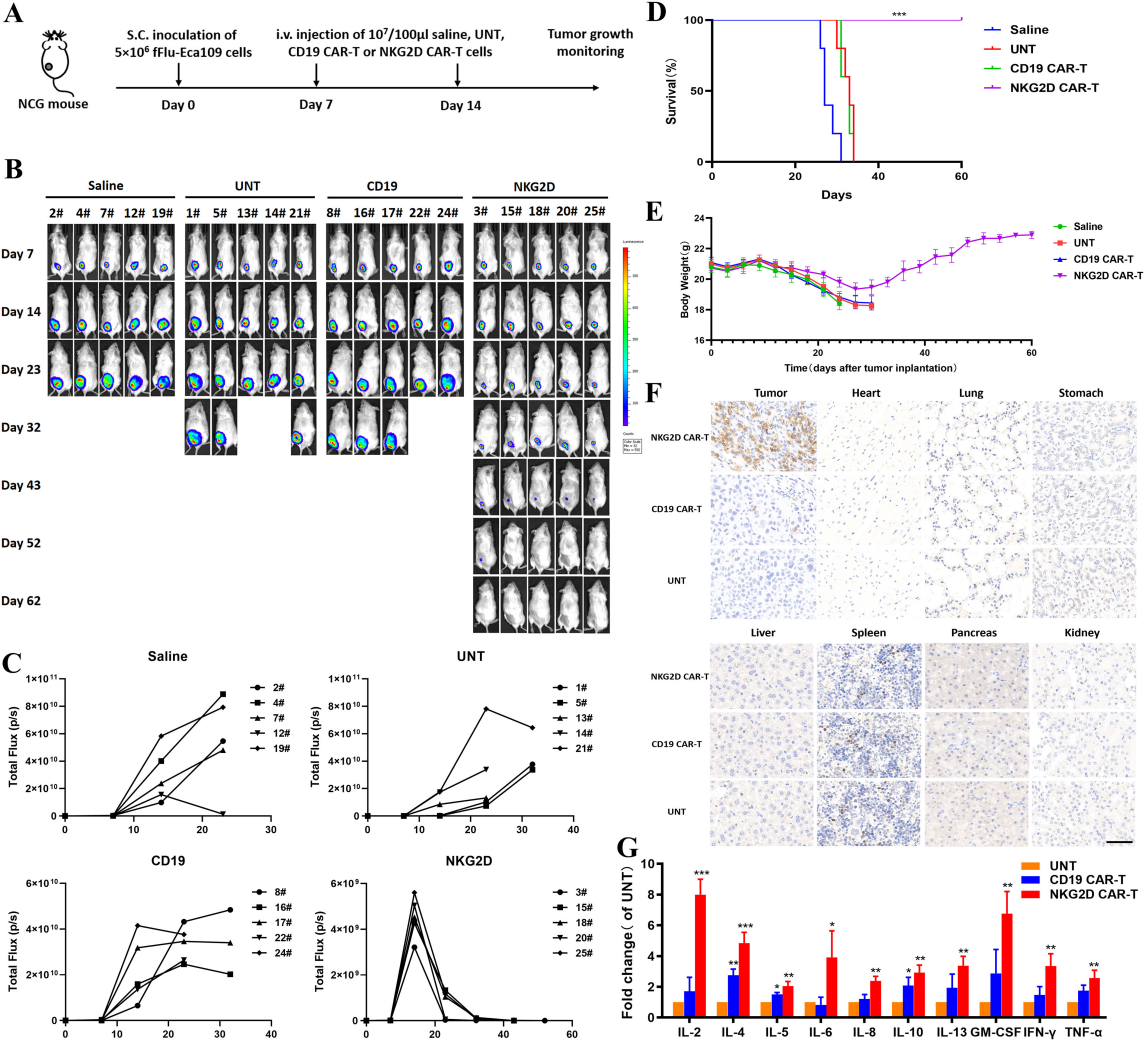
Effects of NKG2D CAR-T cells in a human ESCC Eca109 xenograft model. **(A)** The experimental outline of the animal study. Four groups of NCG mice (n=5) received a subcutaneous inoculation of 5x10^6^ Eca109-fLuc cells (day 0) followed by two intravenous injections of saline, UNT, CD19 CAR-T cells or NKG2D CAR-T cells on days 7 and 14 (1x10^7^ cells/100 µl per injection per mouse). **(B)** Images of the tumor burden by bioluminescence imaging from day 7 to day 62 after tumor implantation. **(C)** Growth curve of Eca109-fLuc xenografts treated as described above. **(D)** The survival of mice was monitored up to day 62 after tumor implantation and analyzed via the Kaplan–Meier method. Statistical analysis of survival between groups was performed via the log-rank test, ***P < 0.001. **(E)** Body weight changes were monitored every three days over the course of survival. **(F)** Representative images of HE (hematoxylin and eosin) staining of tumors and major organs (heart, lung, stomach, liver, spleen, pancreas and kidney) from NCG mice treated with UNT, CD19 CAR-T or NKG2D CAR-T cells. Tumors and major organs were collected for immunohistochemical analysis 5 days after T cells infusion. Formalin-fixed, paraffin-embedded tumor sections were cut and stained for human CD3 expression (brown); scale bar=50 μm. **(G)** The cytokine levels of IL-2, IL-4, IL-5, IL-6, IL-8, IL-10, IL-13, TNF-α, IFN-γ and GM-CSF in the circulation of NCG mice after T cells administration. The peripheral blood of NCG mice was harvested 5 days after the second T cell infusion for Luminex liquid suspension chip detection. *P < 0.05, **P < 0.01, ***P < 0.001.

We generated a human ESCC xenograft model by subcutaneously injecting Eca109-fLuc cells into NCG mice to evaluate the antitumor effects of NKG2D CAR-T cells *in vivo*. Four groups of NCG mice were intravenously administered saline, UNT, CD19 CAR-T, or NKG2D CAR-T cells on days 7 and 14 after tumor implantation (**Figure 4A**). Tumor growth was monitored through bioluminescence imaging at the indicated times. ESCC xenografts grew rapidly in all the mice treated with saline, UNT, or CD19 CAR-T cells, resulting in animal death or euthanasia due to being moribund around day 30. In contrast, tumors treated with NKG2D CAR-T cells progressively decreased and gradually shrank to disappearance, with no tumor recurrence observed until day 62 after tumor implantation, the longest time point monitored in the experiment (**Figures 4B–D**). Additionally, the animals treated with NKG2D CAR-T cells showed no significant body weight loss or other xenogeneic graft-versus-host disease (x-GVHD) symptoms compared with those in the CD19 CAR-T, UNT cells and saline-treated groups (**Figure 4E**).

In addition, we collected xenograft tumors and major organs from the three groups of T cell-treated mice to evaluate the accumulation and persistence of human T cells *in vivo*. Immunohistochemical analysis was performed using an anti-CD3ζ antibody. Abundant human T cells were found in the xenografts of mice treated with NKG2D CAR-T cells, whereas only a few T cells were detected in those treated with UNT or CD19 CAR-T cells (**Figure 4F**). No human T cells or organic lesions were detected in the major organs of any of the three groups. The results illustrated the safety of NKG2D CAR-T cells therapy as well as the preferential localization and persistence of NKG2D CAR-T cells in ESCC xenografts.

The peripheral blood of the mice was harvested 5 days after the second T cell infusion to assess the levels of 10 cytokines, including IL-2, IL-4, IL-5, IL-6, IL-8, IL-10, IL-13, TNF-α, IFN-γ, and GM-CSF. The results revealed significantly higher cytokine levels in the circulation of NKG2D CAR-T cells treated mice than in that of UNT or CD19 CAR-T cells treated mice, suggesting the specific immunological activity of NKG2D CAR-T cells toward Eca109 xenografts *in vivo* (**Figure 4G**). These results demonstrate that NKG2D CAR-T cells specifically homed to and accumulated in ESCC tumors and exerted efficient immunological activity correlating with tumor regression.

### 3.5 Preconditioning with irradiation enhances the efficacy of NKG2D CAR-T cells in treating ESCC xenograft models

**Figure 5:**
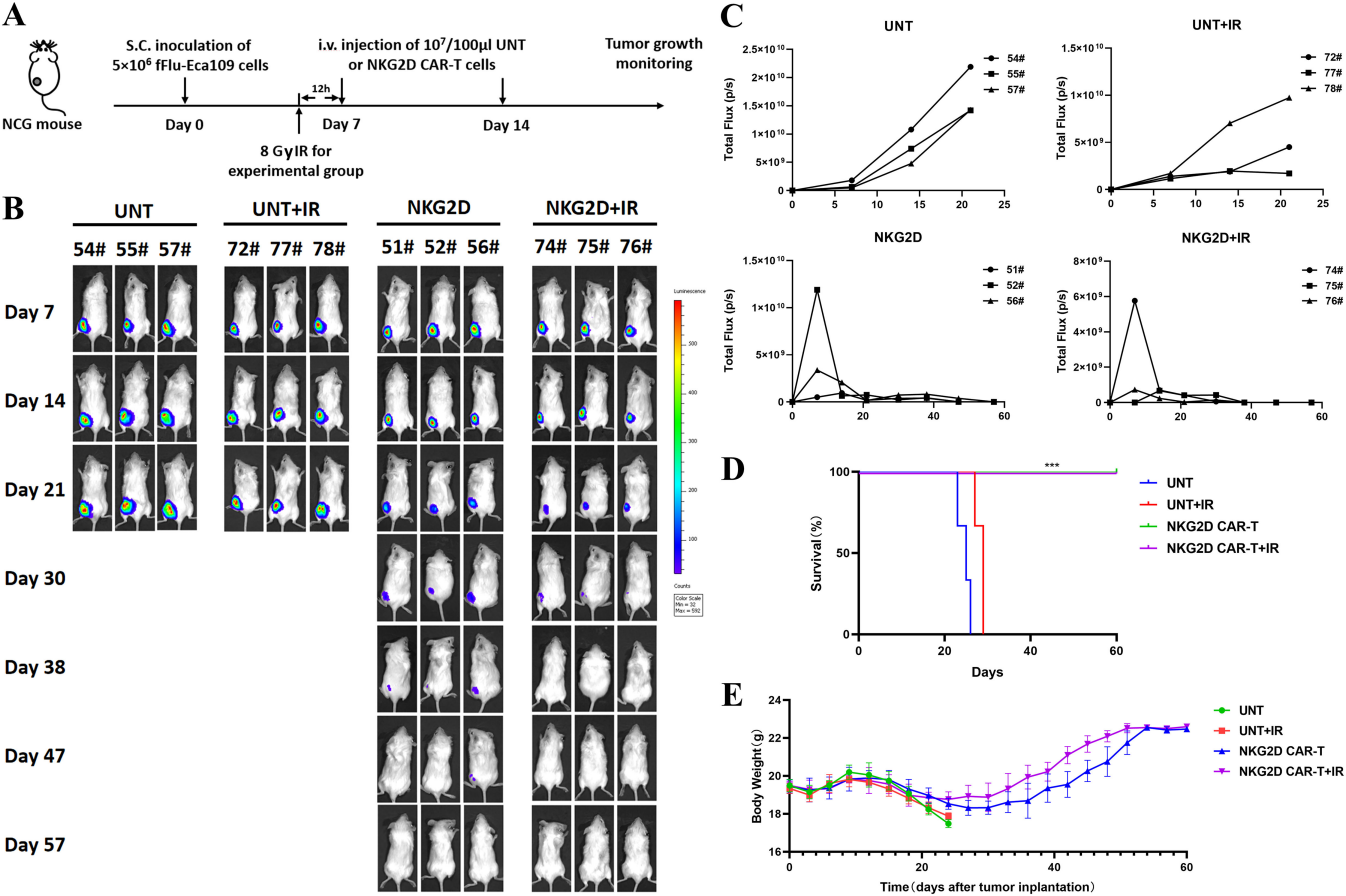
Combination of radiotherapy and NKG2D CAR-T cells in a human ESCC Eca109 xenograft model. **(A)** The experimental outline of the animal study for combined therapy. Four groups of Eca109 tumor-bearing NCG mice (n=3) were treated intravenously with UNT or NKG2D CAR-T cells on days 7 and 14 after tumor implantation (1x10^7^ cells/100 µl per injection per mouse). A single local irradiation of 8 Gy was performed 12 hours prior to T cells administration in the combination treatment group. **(B)** Images of the tumor burden by bioluminescent imaging from day 7 to day 57 after tumor implantation. **(C)** Growth curve of Eca109-fLuc xenografts treated as described above. **(D)** The survival of mice was monitored up to day 57 after tumor implantation and analyzed via the Kaplan–Meier method. Statistical analysis of survival between groups was performed via the log-rank test, ***P < 0.001. **(E)** Body weight changes were monitored every three days over the course of survival.

Based on the *in vitro* experimental results that irradiation enhanced the function of NKG2D CAR-T cells against ESCC cells, we established the same ESCC subcutaneous xenograft model as above to evaluate the potential of combined treatment with radiotherapy and NKG2D CAR-T cells for ESCC *in vivo*. NCG mice were treated with two infusions of UNT or NKG2D CAR-T cells, either alone or in combination with local irradiation. The mice in the combination treatment group were preconditioned with a single dose of 8 Gy irradiation 12 h prior to T cells administration (**Figure 5A**). Local radiotherapy combined with UNT cells had no obvious effect on tumor regression or survival duration of mice, while radiotherapy combined with NKG2D CAR-T cells showed a potential trend toward accelerated tumor shrinkage compared to CAR-T monotherapy (**Figures 5B, C**), though further validation in larger cohorts is required. However, it is regrettable that we did not observe a difference in overall survival between the two NKG2D CAR-T cells treated groups, as all the mice were cured with either CAR-T cells alone or the combination treatment (**Figure 5D**). Long-term observation or rechallenge with live tumor cells for memory responses may highlight more notable benefits of the combined therapy. There were no noticeable differences in body weight or relevant symptoms among the four groups of mice. (**Figure 5E**). To some extent, these results indicate that local radiotherapy has the potential to enhance the antitumor function of NKG2D CAR-T cells against ESCC xenografts *in vivo* without raising additional toxicity.

### 3.6 Local radiotherapy alters the TME and promotes the migration of NKG2D CAR-T cells into tumor sites

**Figure 6:**
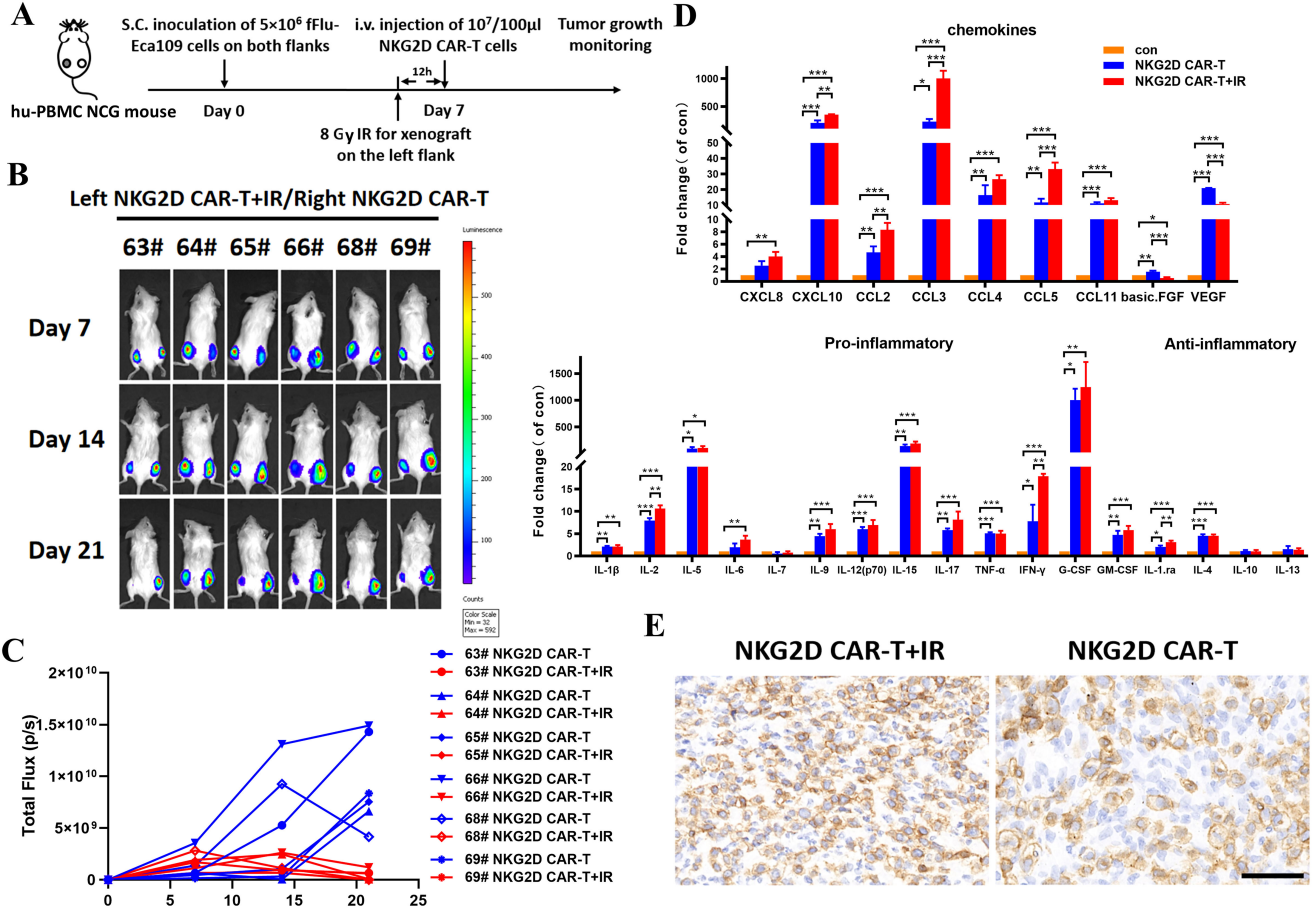
The role of local radiotherapy for TME alteration and NKG2D CAR-T cells infiltration into tumor sites in a bilateral ESCC xenografts model. **(A)** The experimental outline of the bilateral ESCC xenografts study. Hu-PBMC NCG mice (n=6) were subcutaneously injected with 5x10^6^ Eca109-fLuc cells on both flanks to establish a bilateral ESCC xenografts model. Local radiotherapy with a single dose of 8 Gy was performed toward xenografts on the left side 12 hours before T cells injection, while xenografts on the right side received no additional treatment. NKG2D CAR-T cells were injected intravenously on day 7 after the bilateral tumor implantation (1x10^7^ cells/100 µl per injection per mouse). **(B)** Images of the tumor burden by bioluminescence imaging from day 7 to day 21 after tumor implantation. **(C)** Growth curve of Eca109-fLuc xenografts on both sides of mice treated as described above. **(D)** The multiple cytokine levels in the bilateral tumor tissue of NCG mice treated with NKG2D CAR-T cells combined with irradiation or not, tumors without any treatment were used as control. Bilateral xenografts were harvested 5 days after T cells infusion, proteins in the tumor tissue were extracted, and cytokines were evaluated by Luminex liquid suspension chip detection. *P < 0.05, **P < 0.01, ***P < 0.001. **(E)** Representative images of immunohistochemical staining of the bilateral tumors treated with NKG2D CAR-T cells combined with irradiation or not. Xenografts were collected from NCG mice 5 days after T cells infusion. Formalin-fixed, paraffin-embedded tumor sections were cut and stained for human CD3 expression (brown); scale bar = 50 μm.

In addition to the upregulated expression of NKG2DLs in tumor cells, the influence of irradiation on the tumor microenvironment (TME) may also account for the enhanced effects of NKG2D CAR-T cells on ESCC xenografts. Therefore, we established a bilateral Eca109 xenograft model in hu-PBMC NCG mice. NKG2D CAR-T cells were injected intravenously on day 7 after matching tumor implantation in both flanks. The tumor on the left side received local irradiation of 8 Gy 12 hours before T cells injection, whereas the tumor on the right side received no irradiation to serve as a control (**Figure 6A**). Bioluminescence imaging revealed that preirradiated tumors on the left side were significantly inhibited after NKG2D CAR-T cells administration compared with contralateral tumors in the same mice (**Figures 6B, C**).

We then collected bilateral xenografts for Luminex liquid suspension chip detection to verify the changes in cytokines and chemokines at the irradiated tumor sites. The results revealed that irradiation increased the levels of chemokines involved in the migration of T cells (e.g., CXCL10, CCL2, CCL3, and CCL5) but decreased the levels of proangiogenic factors (e.g., VEGF and basic FGF), which facilitate T cells infiltration into the tumor bed (**Figure 6D**). In addition, local radiotherapy resulted in inflammatory TME remodeling with increased levels of proinflammatory factors (e.g., IL-2, IL-6, IL-9, IL-15, IL-17, IFN-γ, G-CSF, and G-CMSF), although the difference was not significant (**Figure 6D**). Furthermore, immunohistochemical analysis revealed preferential T cells infiltration in preirradiated tumor tissues (**Figure 6E**). These results demonstrated the role of local irradiation in promoting the migration of NKG2D CAR-T cells to tumor sites. Collectively, a single local irradiation with 8 Gy was sufficient to enhance the antitumor effects of NKG2D CAR-T cells against ESCC xenografts, by effect of upregulating NKG2DL expression on the surface of tumor cells, as well as altering the TME, thereby promoting the migration and infiltration of NKG2D CAR-T cells into tumor sites.

## 4 Discussion

CAR-T cells therapy has shown remarkable therapeutic efficacy for hematological malignancies; however, challenges remain for its application in solid tumors. The challenges that need to be addressed include the high heterogeneity of solid tumor cells, paucity of targeted antigens, restricted migration and persistence of T cells at the tumor site, immunosuppressive TME, on-target off-tumor toxicity, and integration with conventional anticancer treatments (26). To date, the exploration of CAR-T cells therapy in esophageal cancer has been limited, with only EphA2, HER2, MUC1, and CD276 being identified as potential therapeutic targets in preclinical studies.

Several advantages have been demonstrated for NKG2D CAR compared with other classical CARs. First, NKG2D CAR-T cells can target multiple ligands expressed on tumor cells and are thus beneficial for overcoming the antigen escape of traditional CAR T cells that target only a single antigen. Additionally, NKG2DLs are detected not only in tumor cells but also in regulatory T cells, myeloid cells, and endothelial cells in the TME (27). Several preclinical studies have demonstrated the potential of NKG2D CAR-engineered T cells or NK cells to recruit and boost endogenous anti-tumor immune cells, prevent tumor neovasculature, and target myeloid-derived suppressor cells or regulatory T cells, thereby ameliorating the inhibitive TME (28–32).

In this study, we first demonstrated that NKG2D CAR-T cells were able to specifically recognize and kill NKG2DL-expressing ESCC cell lines *in vitro*, and the immunological response was dose-dependent and NKG2D signal-dependent. We then verified the therapeutic efficacy, as well as the accumulation and persistence of NKG2D CAR-T cells in a human ESCC xenograft model. Furthermore, we revealed an enhanced cytotoxic effect of NKG2D CAR-T cells on preirradiated ESCC cells both *in vitro* and *in vivo*, in view of increased NKG2DLs expression and altered TME upon irradiation. While no advantage in overall survival was observed during the limited follow-up period, the accelerated tumor shrinkage in mice treated with the combined therapy presents an opportunity for further study to investigate and demonstrate more notable effects of combining radiotherapy with NKG2D CAR-T cells for ESCC.

The combination of CAR-T cells therapy with other treatments has revealed promising prospects for enhancing the efficacy of cancer therapies (33). Radiation can provoke a systemic immune response and reprogram the TME from immunosuppressive “cold” to immune-inflamed “hot” (34). Radiation can also increase antigen-presenting cell (APC) activation, human leukocyte antigen (HLA), and target antigen display on tumors to improve tumor recognition (35). Many studies have characterized the reprogrammed TME and improved tumor recognition induced by radiotherapy to support the potential of radiotherapy to synergize with CAR-T cells therapy (36, 37). Here, we clarified the effect of local radiotherapy combined with NKG2D CAR-T cells in a bilateral ESCC xenograft model, as demonstrated by increased chemokine levels and more T cells infiltration in preirradiated xenografts, and consequently enhanced anti-tumor efficiency.

Several advantages of the combination strategy are considered. First, pretreatment of tumors with local radiotherapy leads to increased NKG2DL exposure, an altered TME, preferable T cells infiltration at tumor sites, and consequently better tumor control caused by NKG2D CAR-T cells, while yielding a very low radiotoxicity burden. Besides, NKG2D CAR-T cells treatment could have a potential role in eradicating minimal residual disease and micrometastatic disease in ESCC patients, as CAR-T cells can survive and generate a population of long-lived central memory T cells for long-term immunological surveillance (38). Scientists have been making efforts to increase CAR-T cells expansion and memory-like cell formation, reduce exhaustion, and support the persistence of CAR-T cells *in vivo*, aiming to improve the outcomes of CAR-T cells therapy (39–42). In addition, as the expression of NKG2DLs can be induced by many pathways, NKG2D CAR-T cells have been investigated in combination with various reagents, such as histone deacetylase inhibitors, Dickkopf-1 inhibitors, or chemotherapeutic agents, for synergistic and superior antitumor effects (10, 12, 43). This poses a potential risk of on-target, off-tumor toxicity, since systemically administered reagents may also increase NKG2DL expression in healthy tissues. However, local radiotherapy combined with NKG2D CAR-T cells would not raise this type of risk, as the effect of radiation is restricted to the target tumor region. Nevertheless, more mechanistic explorations are needed to understand and guide how the combined treatment will best work, such as the appropriate dose or fractionation scheme of radiotherapy and the best schedules for radiotherapy and CAR-T cells administration, which will enable the design of more effective combinations of radiation and NKG2D CAR-T cells (44).

There are several limitations of our study to be considered. First, while our findings demonstrate the efficacy of NKG2D CAR T cells across three distinct preclinical models, independent repetition of the *in vivo* studies should be conducted to further validate the robustness of these results, which is vital for drawing rigorous conclusions; and long-term observation or rechallenge with live tumor cells are needed for further study to highlight OS difference between therapy of NKG2D CAR T with or without radiotherapy. Second, the use of NCG and Hu-PBMC NCG mouse model restricts our ability to comprehensively assess the impact of radiotherapy on the tumor microenvironment, as these mice do not harbor the complete repertoire of immune cells that contribute to immunosuppression. Third, local radiotherapy in the bilateral ESCC xenografts model did not elicit systemic abscopal effects on non-irradiated tumors, likely due to the absence of fully functional immune system, and the suboptimal radiation dosing (8 Gy single fraction) or inherent radioresistance in the ESCC. Finally, the immunomodulatory effects of radiotherapy on the TME are complicated, and highly dependent on dose fractionation regimens and tumor immunogenicity; while our demonstration of TME alteration was limited, focusing mainly on cytokines and T cells infiltration. Future work should employ spatial transcriptomics and multiplex IHC to map the stromal-immune crosstalk, in order to enhancing our insights into radioimmunotherapy.

NKG2D CAR-T cell therapy represents a promising immunotherapeutic approach for cancer treatment, demonstrating notable clinical efficacy in early-phase trials (45). Recent research has revealed the potential of NKG2D CAR-T cells in eliminating senescent cells, which often accumulate in the TME and contribute to tumor progression (46, 47). This novel finding adds a unique dimension to the therapeutic mechanism of NKG2D CAR-T cells. The future of NKG2D CAR-T therapies also presents several challenges that need to be addressed. One critical concern is the potential for “fratricide,” where NKG2D CAR-T cells may attack one another due to low-level expression of NKG2DLs on activated T cells. Researchers have explored solutions like PI3K inhibitors, blocking antibodies or adaptor-based structure to control fratricide and enhance the stability and persistence of CAR-T cells in culture (48). Another challenge is the risk of immune evasion caused by the shedding of soluble NKG2DLs, which can block NKG2D-mediated immune activation. Strategies such as antibody-mediated inhibition of proteolytic enzymes, or agents that upregulate NKG2DLs expression such as HDAC inhibitors or proteasome inhibitors, have shown potential in preclinical models (4, 5). Furthermore, NKG2D CAR engineered NK cells offer a compelling alternative with lower risk of CRS and neurotoxicity compared to T cell-based platforms. Advances in cellular engineering, precision CAR design, NKG2DLs signal enhancement, and synergistic combinations will be critical to maximizing the therapeutic potential of NKG2D CAR-T or CAR-NK cells across solid and hematologic malignancies.

## 5 Conclusion

Taken together, this study clarified the therapeutic potential of NKG2D CAR-T cells in preclinical ESCC models, and demonstrated the role of local radiotherapy in improving the effectiveness of NKG2D CAR-T cells therapy. Our findings suggest that NKG2D CAR-T cells in combination with radiotherapy offer a potentially curative therapeutic approach for ESCC patients, especially those who are unable to undergo resection or full-dose radiotherapy.

## Data Availability

The original contributions presented in the study are included in the article/supplementary material. Further inquiries can be directed to the corresponding authors.
